# Semen collection, evaluation, and cryopreservation in the bonobo (*Pan paniscus*)

**DOI:** 10.1186/s40850-022-00110-3

**Published:** 2022-02-10

**Authors:** Ilse Gerits, Eline Wydooghe, Sofie Peere, Francis Vercammen, Jeroen M. G. Stevens, Cyriel Ververs

**Affiliations:** 1 Department of Reproduction, Obstetrics and Herd Health, Faculty of Veterinary Medicine, Ghent University, Salisburylaan 133, 9820 Merelbeke, Belgium; 2 Antwerp Zoo Centre for Research and Conservation, 2000 Antwerp, Belgium; 3 Behavioral Ecology and Ecophysiology Group, Department of Biology, Antwerp University, 2000 Antwerp, Belgium; 4 SALTO – Agro and Biotechnology Odisee University College, 9100 Sint Niklaas, Belgium

**Keywords:** Bonobo, Cryopreservation, Electro-ejaculation, Evaluation, Extenders, *Pan paniscus*

## Abstract

**Background:**

Captive breeding of bonobos (*Pan paniscus)* has proven to be successful, but maintaining genetic diversity remains a challenge. Cryopreservation of semen is an important potential tool to maintain genetic diversity by preserving current genetic material for future use, as well as facilitating the transport and exchange of genetic material. This study aimed to develop a protocol for semen collection and cryopreservation in the bonobo. Semen was collected from four healthy adult bonobos under general anesthesia during management translocation procedures. Semen collection utilizing urethral catheterization was not successful (*n* = 1), however, all males (*n* = 4) responded well to rectal probe electro-ejaculation. Immediately after collection, ejaculates were evaluated for color and admixtures, volume, motility, and concentration. Eosin-Nigrosin staining was prepared to evaluate morphology and viability. Ejaculates were split into two equal volumes and cryopreserved in two different extenders, using a one-step and a two-step approach. Ejaculates were gradually cooled to 4 °C in two hours, subsequently stored in liquid nitrogen vapor for twenty minutes (0.25 ml straws), and finally dropped into liquid nitrogen.

**Results:**

Pre-freeze evaluation showed thick, white samples with an average ejaculate volume of 450 µl (100-1000 µl), total motility of 59% (40–80%), viability of 69% (38–85%) and 58% (46–72%) normal spermatozoa. Mainly head (22%) and tail (19%) defects were detected on the Eosin-Nigrosin stain. Ejaculates were highly concentrated, nevertheless, due to the coagulum that caused high viscosity and non-homogenous fractions, only estimations of concentration could be made (1000 million/ml). After 24 h of storage, the post-thaw evaluation showed a loss of quality with an average post-thaw total motility of 15% (5–25%) using the one-step freezing medium, and 19% (5–30%) using the two-step medium. Average post-thaw viability was 15% (4–24%) and 21% (15–29%), respectively.

**Conclusions:**

This report on ejaculates from bonobos obtained by rectal probe electro-ejaculation shows that semen parameters of this species are not completely similar to those of its sibling species, the chimpanzee. Further studies are necessary to develop an optimal protocol for the processing and cryopreservation of bonobo spermatozoa.

## Background

Bonobo population in the wild is rapidly declining, mainly due to poaching and habitat loss, with global climate change and mortality due to contagious diseases listed as additional potential risks [1–3]. For this reason, in 1996 the bonobo was classified as "endangered" on the International Union for Conservation of Nature's (IUCN) Red List [1].

In addition to protecting the species in its natural habitat by land protection, creation of nature reserves, and law enforcement, captive populations can serve as a "back-up" population, and maintain a reservoir of genetic diversity. An international studbook contains the registration number of each animal of a particular species kept under human care, its sex and birthdate, the identity of its parents, where it was born, and where (and when) it was transferred to other institutions. Antwerp Zoo currently maintains a studbook for all captive bonobos with 212 individuals currently being monitored across Europe by the European Association of Zoos and Aquaria (EAZA) Ex Situ Program (EEP), and 90 being monitored across the United States by the American Zoo and Aquaria Association (AZA) Species Survival Program (SSP) [4]. This globally managed population has 36 founder species that are not closely related and originate from different areas within the bonobo natural range [2, 5, 6]. Due to this limited founder number and skewed founder representation, both programs (EEP and SSP) collaborate extensively to maintain genetic diversity. Balancing founder representation can be challenging in combination with the multimale-multifemale social organization and promiscuous mating system of this species [7–9]. To mimic the sociosexual behavior of wild bonobos, captive programs aim to keep males in their natal group and transfer females between zoos when they become adolescents. However, keeping multiple breeding males in the group is hard in preventing the genetically overrepresented males in that group from breeding and encourages the genetically underrepresented males to breed [8, 10, 11]. This sometimes necessitates transferring males from their natal group in light of increasing the genetic diversity. Artificial insemination (AI) using cryopreserved semen can reduce the number of male transfers and will lead to a more equal representation of the founder genetic material without disturbing the social organization within the population [3, 5, 10]. Despite the potential importance of AI for captive breeding programs, the largest experience base with AI in primates is in humans. To date, only a few published reports describe live birth after insemination of frozen semen in non-human primates [12]. To obtain a successful AI protocol, multiple aspects and procedures have to be considered both in males and females [13]. This study will focus on the semen collection method, semen characteristics, and cryopreservation of male bonobo gametes.

At present, very little experience with AI in bonobos exists. Sperm collection, freezing the semen in liquid nitrogen, intercontinental transfer of frozen sperm and attempts to inseminate female bonobos in zoos have occurred as early as the 1970s and 1980s [12, 14]. However, these attempts are merely based on very small sample sizes without detailed protocols [12, 14, 15] and so far there are no reports on successful confirmed artificial insemination in this species. We aimed to fill this gap of knowledge by reporting on a case study where four male bonobos within the same institution were anesthetized for translocation within the same facility. This created an opportunity to investigate two methods for semen collection being rectal probe electro-ejaculation and urethral catheterization, to describe physiological parameters of sperm (both fresh and thawed) and to test two protocols for cryopreservation.

To date, different techniques for semen collection have been described in great apes. Naturally-ejaculated sperm can be obtained by masturbation, the use of an artificial vagina, or vaginal flush after copulation [16–20]. Although the quality of naturally-ejaculated semen is superior compared to ejaculates obtained after electro-ejaculation, it is a challenge to obtain naturally-ejaculated, uncontaminated semen from non-human primates [16]. These procedures require a level of training of the animals, which is not the case for the bonobos housed in this institute. Although postmortem semen collection has been described to be successful in the bonobo [21], this method cannot be attempted in this study.

Electro-stimulation, both rectal probe, and direct penile stimulation, has been described as a rewarding method for semen collection in non-human primates [16, 17, 19, 21]. It is known that rectal probe electro-ejaculation yields lower quantities and qualities of ejaculates in chimpanzees, compared to live masturbation [16, 17, 19]. Stimulation of the accessory sex glands during the procedure of electrostimulation will affect the composition of the seminal plasma, which can explain the lower quality of electro-ejaculated spermatozoa [16, 19]. However, a sperm penetration assay on chimpanzee semen showed no significant difference in the fertilizing capacity of spermatozoa obtained by rectal probe electro-ejaculation compared to live masturbation [16, 17]. Unfortunately, no standardized protocol for rectal probe electro-ejaculation is available and the requirement for specific equipment and a trained operator are the limitations of this technique [22, 23]. Another semen collection method described in wildlife species is urethral catheterization [24–30], but this method has, to our knowledge, not been described yet in primates. Urethral catheterization is based on the α-adrenergic effect of the anesthesia which initiates contractions of the ducti deferentes. By catheterization of the urethra, the tip of the catheter is placed at the position of the prostate and a semen sample can be obtained by capillarity [24, 31]. Urethral catheterization is an easy and less invasive technique that will be explored as an alternative next to rectal probe electro-ejaculation performed in this study.

In most domestic species there is a correlation between testicle size, scrotal width or circumference on one hand, and total semen output on the other hand [13]. We measured the testicular size and bodyweight of the studied bonobos because comparative information of these parameters is not yet available in bonobos [32]. Semen processing and cryopreservation is a routine method in humans, but extensive information on semen characteristics and processing in non-human primates is missing in most species. We used conventional semen characteristics to estimate fertility potential, including semen volume, sperm concentration, motility, live/death ratio, and morphology. For cryopreservation of non-human primate sperm achieved by rectal probe electro-ejaculation, 5% glycerol as a cryoprotectant is considered to be optimal, although other cryoprotectants such as DMSO have been described in other cryopreservation protocols [16]. In this study, two semen extenders for cryopreservation were compared that slightly differ in composition. Both extenders are egg-yolk-based extenders containing gentamycin as an antibiotic but the main difference is the variation in the final concentration of glycerol. A one-step cryopreservation medium with 12% glycerol was compared to a two-step medium with 5% glycerol. In the two-step medium, the sperm cells were gradually exposed to the glycerol: in the first step 3% of glycerol is used and in the second step 7%, to end with a final concentration of 5% glycerol [33, 34]. As a buffer, the one-step medium contains N-tris(hydroxymethyl)-two-aminoethane sulfonic acid (TES) and Tris(hydroxymethyl)-aminomethane (Tris) (TEST-yolk buffer) whereas the two-step medium contains only Tris. The two-step medium is prepared in-house, while the one-step medium is manufactured as ready-to-use.

We can conclude that conservation of the bonobo would benefit from detailed protocols for artificial reproductive techniques. We hope this study includes valuable information for researchers in this domain.

## Results

### Semen collection

The average testicle size of the four studied adult bonobos showed less than 10% inter-testicular difference between the animals (Table 1). The testicle diameter, as well as the total scrotal circumference, was not positively correlated with the bodyweight of the animals (Table 1).

Semen collection utilizing urethral catheterization was not successful (*n* = 1). However, all adult males (*n* = 4) responded well to rectal probe electro-ejaculation. Erection during rectal probe electro-ejaculation occurred around pulses of 2 to 3 V (V), and ejaculation started at 3 V on average (Table 1). Electric pulses were given up to 9 V depending on the reaction of the animal.

**Table 1:** Overview of some general characteristics, the results of the measurements of the testicular size, and the semen characteristics (pre-freeze and post-thaw) of each individual (n1 – n4)

	n1		n2	n3		n4	
Date of birth	16/04/1998		19/04/1998	23/07/1994		29/01/2006	
Weight (kg)	38.55		39.75	38.70		33.70	
Left testicle (cm)	6.0 × 3.6		7.8 × 5.1	5.6 × 4.0		7.0 × 4.5	
Right testicle (cm)	5.5 × 4.0		7.6 × 5.5	5.5 × 4.5		7.0 × 4.3	
Total scrotal circumference (cm)	24		30	25		27	
Volume of semen (µl)	400		100	300		1000	
Amount of diluter (µl)	2000		500	1600		5000	
Number of cycles	2		3	3		4	
Voltage at semen emission (V)	3.0–4.0		4.0–5.0	3.0		3.0–5.0	
Pre-freeze (%)							
Total motility	42		40	75		80	
Live	85		38	83		69	
Dead	15		62	17		31	
Morphology (%)							
Normal	46		46	69		72	
Abn. head	18		22	22		26	
Abn. tail	36		32	9		1	
Prox. prot. dropl	0		0	0		1	
Dist. prot. dropl	0		0	0		0	
Post-thaw (%)	1-step medium	2-step medium	2-step medium	1-step medium	2-step medium	1-step medium	2-step medium
Total motility	< 5	5	15	25	30	12	26
Live	4	16	15	24	29	16	25
Dead	96	84	85	76	71	84	75

### Semen evaluation and cryopreservation

The samples collected by the use of rectal probe electro-ejaculation had an average volume of 450 µl (Table 1). Admixtures due to urine or red blood cell contamination were not noticed. Ejaculates were highly concentrated due to high viscosity, low volumes, and non-homogenous samples. Therefore, only estimations of concentration could be made (around 1000 million/ml). Due to the coagulum in the semen, samples were found non-homogenous.

**Fig. 1:**
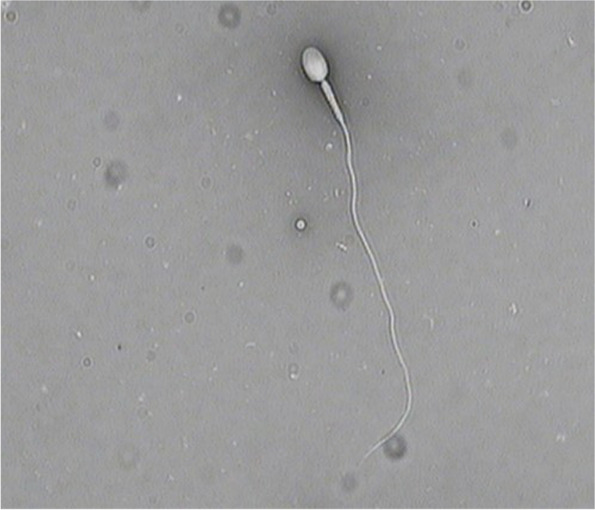
Normal spermatozoon

Microscopically the total motility, morphology, and viability were assessed in the pre-freeze and post-thaw The pre-freeze evaluation showed thick, white samples and on average total motility of 59% (40–80%), the viability of 69% (38–85%), and 58% (46–72%) normal spermatozoa (Fig. 1). Mainly head (22%) and tail (19%) defects were detected on the Eosin-Nigrosin stain. After 24–48 h of storage, the post-thaw evaluation showed a loss of quality with an average post-thaw total motility of 15% (5–25%) using the one-step freezing medium, and 19% (5–30%) using the two-step medium. Average post-thaw viability showed 15% (4–24%) and 21% (15–29%) respectively. For n2, only the two-step medium was used because of the low sample volume (Table 1).

## Discussion

### Semen collection

This study showed that semen collection using rectal probe electro-ejaculation under general anesthesia was successful in all four adult bonobos. Electric pulses were given up to 9 V (4,5 V-9 V) depending on the reaction of the animal, such as muscular contractions in the hind limbs, erection and ejaculation. Wherein in this study, all animals obtained an erection, which contrasts with previous primate studies where a gradual increase to 12 V did not result in an erection [12, 14]. In other studies on rectal probe electro-ejaculation in non-human primates, stimulating parameters vary widely (2 V-18 V), but in general, a higher frequency and more pulses per series were used [14], compared to this study. Besides the stimulation of the hypogastric and internal pudendal nerves needed for the erection and ejaculation, the stimulation by rectal probe electro-ejaculation might also lead to unwanted muscular contractions of the hind legs. These contractions were observed in all four bonobos in this study when stimuli were given at 5 V or higher. In humans, rectal probe electro-ejaculation is used to collect semen in patients with anejaculation after spinal cord injury and these patients do not need analgesics or anesthetics. In contrast, men with normal sensation in the perirectal area are given general or epidural anesthesia before electro-ejaculation because the procedure can lead to severe discomfort [35, 36]. In our study, the heart rate of all four bonobos was monitored closely during the whole procedure and no increase or abnormalities could be detected utilizing echocardiographic examination. After finishing the procedure, the bonobos were closely monitored for the next 48 h by a team of keepers, ethologists, and the zoo veterinarian and research staff. The animals recovered without any problems and showed no apparent change in appetite or behavior in the following hours and days. Considering these parameters, we believed that rectal probe electro-ejaculation under general anesthesia, as part of routine management procedures, has no detrimental effect on the bonobo well-being [11].

Urethral catheterization offers a safe, easy and fast alternative to rectal probe electro-ejaculation for semen collection, for example in domestic dogs and cats as well as in some wild feline and canine species [24, 34]. Unfortunately, semen collection by urethral catheterization attempted in one bonobo, was not successful in this study. This may be due to the coagulum in the semen or to other aspects of the anatomy of the male reproductive tract, for example the length of the urethra of the bonobo is unknown. As urethral catheterization is based on the α-adrenergic effect of the anesthesia which initiates contractions of the ducti deferentes [24, 34], the anesthetic protocol and drugs dosages used in the current study could interfere and contribute to this method not being successful. Further exploration of this technique in non-human primates is necessary, and ultrasound guidance of the male reproductive tract during the catheterization process could provide useful insights into whether this technique can be applied to primates.

### Semen evaluation

The sample size in this study is arguably small. Bonobos are only kept in zoos and have rarely been housed in laboratories like chimpanzees, where information on chimpanzee sperm could be collected in larger numbers [17, 19]. The population of bonobos is few in zoos, and typically only a few adult males are housed in the same institute. However, only four adult bonobo males could be investigated in a short period. Due to this small sample size, only descriptive statistics could be performed.

Despite a high viscosity in all semen samples in this study, the ejaculate of two out of four bonobos contained an obvious coagulum (n1 and n2). Although the presence of a coagulum is common in non-human primates [14, 18, 32], previous studies indicate that primate semen may not contain sufficient amounts of proteolytic enzymes to rapidly and automatically liquefy the coagulum in the ejaculate [37–39]. The coagulum normally liquefies at 37 °C [18, 32], yet in this study, this was not the case for bonobo n1. This was also observed for chimpanzee semen where liquefaction of the coagulum only occurred after two to four hours of incubation at 37 °C [17] or after the addition of proteolytic enzymes (e.g. trypsin [14] or type I collagenase [37]). Besides unsatisfactory liquefaction after one hour of incubation, we could observe a deleterious effect on the semen quality (motility, morphology, and viability). In future research, different protocols on bonobo ejaculates should be tested to obtain optimal liquefaction of the coagulum with minimal negative outcomes on sperm quality. Also, a comparison of techniques used in other species with a gelatinous nature of the seminal plasma such as camelids can be useful [32, 33]. Better liquefaction of the ejaculate will probably also affect the ejaculate volume considering the lower volumes of semen samples collected in this study compared to other studies. The bonobo is one of the closest living human relatives, therefore a comparison of human spermatozoa to bonobo spermatozoa can also be meaningful, for example to obtain reference values. Testicle size, scrotal width or circumference could not be correlated with total semen output [13], due to inaccurate measurements of the concentration of the samples.

Pre-freeze motility was very similar to findings in the chimpanzee [14, 19]. In contrast, the bonobo ejaculates in this study showed a higher concentration of morphologically abnormal spermatozoa (41.7 ± 14.2%) than what is described for the chimpanzee (12.2 ± 7.5%) [14, 40]. In humans, semen is known to contain a high proportion (up to 96%) of abnormal spermatozoa [41, 42]. Since bonobos are close relatives to humans, the high proportion of head (22%) and tail (19%) defects observed on Eosin-Nigrosin staining may not be detrimental for the fertilization capacity of these spermatozoa. Head and tail defects can occur as degenerative changes in senescent sperm for example if males have not been serving or masturbating [13]. Artifactual changes can occur simply from cold or osmotic shock, or other handling errors. To decrease the incidence of artifactual Eosin-Nigrosin staining, the slides, stain, and semen must be warm [38, 43–45]. As discussed earlier, the collection method can also influence the quality of the ejaculate [17, 19]. Nevertheless, since the physiological ejaculate parameters of the bonobo are not well studied, future research should focus on the fertilizing capacity of these semen samples.

### Semen cryopreservation

Although the semen cryopreservation protocol used in this study is successfully used in different species [16, 46], we found for both extenders a significant loss of motility and viability of the semen samples after cryopreservation. This loss could be due to the low volume samples and the coagulum in the samples. As a consequence, the samples were cryopreserved based on volume, not based on concentration. For future research, we advise the use of a controlled rate freezer as it is proven that slow, controlled temperature reduction allows better rearrangement of membrane lipids and proteins which is less harmful to cells like spermatozoa [16]. For chimpanzee sperm, a cooling curve of 1 °C per minute from 20 °C to 4 °C, followed by a holding period of 25 min, further cooling to -30 °C at 5 °C per minute and finally cooling to -100 °C at 25 °C per minute was already proven to be successful [16]. For bonobo sperm, no cooling curve has been described yet, so future research should focus on identifying the optimal freezing curves. In the past, bonobo semen cryopreserved in pellet form using a tris diluent with 4% glycerol and 20% egg yolk, thawed in water at 40 °C, showed motility between 10 and 40% [14]. Low volume semen samples in other species have shown good results after vitrification of sperm [31, 45, 47–49]. In this study, the slow-freezing method was used to cryopreserve the semen. Semen vitrification in non-human primates has not been studied yet and should be included in future studies.

## Conclusions

This report on ejaculates from bonobos collected by rectal probe electro-ejaculation under general anesthesia during translocation procedures shows that the parameters of this species are not completely similar to those of its sibling species, the chimpanzee. Further studies concerning semen characteristics, semen collection procedures, ideal semen diluters and optimal freezing protocol are necessary to develop an optimal guideline for the preservation of bonobo semen and the conservation of these endangered species.

## Methods

All of the following described interventions were performed at the request of Planckendael Zoo, a translocation procedure general anesthesia. In this study, four healthy adult bonobo males, born and bred in captivity in two different European zoological institutions were included. At the time of examination they were respectively 21 (= n1), 21 (= n2), 23 (= n3), and 12 (= n4) years old (Table 1). All four bonobos were sexually active (Stevens J.M.G. personal observations) and all of them had produced offspring in the past, proven by DNA testing (n1: four offspring between 06.07.2006 and 17.07.2010; n2: one descendant on 24.03.2012; n3: six offspring between 27.04.2006 and 26.10.2009 and n4: one descendant on 10.04.2016).

Only bonobos that did not want to voluntarily enter the transport box were included in the study. At the start of the procedure, benzodiazepines (midazolam 0.5 mg/kg) were given orally to the bonobo’s and subsequently, they were darted in their enclosure with an alfa-2 agonist (medetomidine 38-44 µg/kg) and dissociative anesthetics (ketamine 3.8–4.4 mg/kg). When the animals were fully anesthetized, an intra-muscular injection of benzodiazepines (diazepam 0.25–0.30 mg/kg) was administered and they were transferred to the examination room. The animals were anesthetized for approximately one hour. First, a general health check was performed, including inspection of teeth and mucosae (oral, nasal, conjunctival), thorax auscultation, and abdominal palpation, as well as a blood sample collection to test for the presence of Simian immunodeficiency virus (SIV), Simian T-cell lymphotropic virus (SLTV), hepatitis B, hepatitis A, herpes simplex and varicella-zoster. In the upper eyelids, an intradermal comparative tuberculin test (with avian and bovine tuberculin) was performed. To end, an echocardiographic examination was carried out by a specialized team of Ghent University for the ‘Great Ape heart project’. After the examination, the animals were placed in individual cages on a large pile of wood wool to provide thermal comfort and were given time to recover from the anesthesia and the procedure. Later on, the same day, when individuals appeared alert, moved around confidently, and responded to keepers’ calls, they were allowed to rejoin close family members first, and the next day joined the entire group to allow for social interactions and social buffering. After the anesthesia, all animals were closely monitored for 48 h in their new enclosure by a team of four experienced keepers, two ethologists, a research assistant, the zoo veterinarian, and the veterinarian assistant. No remarkable changes in behavior, appetite, urination, or defecation were reported, and positive social interactions such as grooming their conspecifics were observed.

### Semen collection

Before starting the procedure, testicles were measured by caliper and tape. To collect a semen sample, two different approaches were tested: urethral catheterization and rectal probe electro-ejaculation. Urethral catheterization was attempted in one bonobo (n1): a catheter (BUSTER®, Disposable Dog Catheter, 2.0 × 500 mm) was inserted into the external urethral opening on top of the penis. Since the length of the urethra is not known, the catheter was inserted four times, each time a bit further to avoid reaching the bladder and contaminating the semen sample with urine. The urinary catheter was inserted 150 mm, 190 mm, 210 mm, and 250 mm during consecutive attempts. During the last attempt, a resistance was noticed without any semen withdrawal into the catheter.

**Fig. 2:**
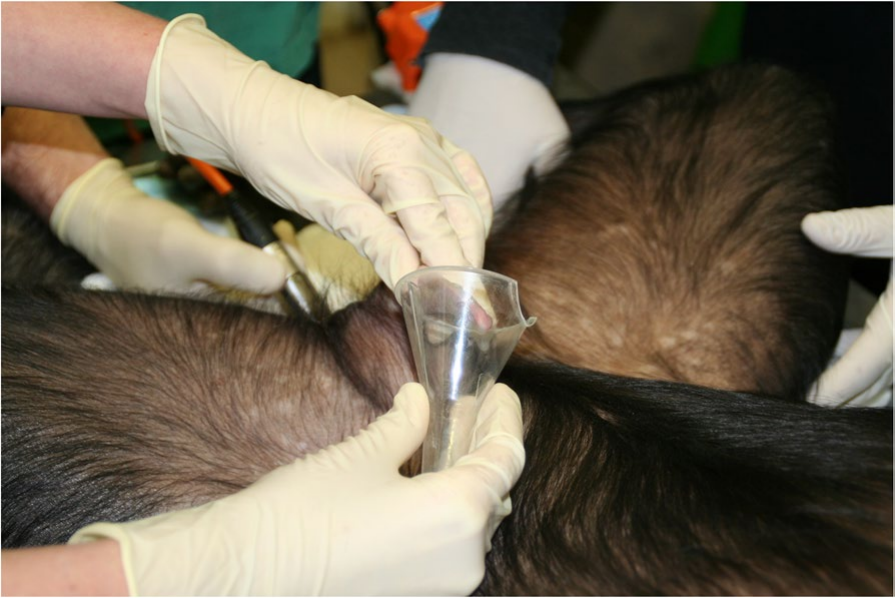
Semen collection. Cranial view of the collection of the ejaculate in a pre-warmed, sterile recipient

Rectal probe electro-ejaculation was performed in all four males, using an Electro Ejaculator from Minitube with a probe 1’’ (Ref. 11,900/0021; 14.5 cm long and 2.6 cm diameter). The feces were removed from the rectum of the bonobo and the rectal probe was positioned into the rectum with the three linear (longitudinal) electrodes directed to the ventral side of the animal [14, 18]. In each bonobo, three or four cycles of electric pulses were completed within a few minutes. One to ten stimuli were given at each voltage, progressing with 0.5-1 V each time, depending on the reaction of the animal such as muscular contractions and the penile response (erection and ejaculation). The first cycle started at 0.5 V, followed by an increase of the starting voltage of 0.5 V for each consecutive cycle. Stimulations varied from 4.5-9 V. The ejaculates were collected in a pre-warmed sterile recipient (Fig. 2) and immediately analyzed macroscopically and microscopically.

### Semen evaluation

Immediately following semen collection, semen quality was assessed. Macroscopic appearance and the parameters volume, concentration, motility, morphology, and viability were evaluated.

The motility and concentration were subjectively estimated by multiple researchers using a phase-contrast microscope with a warming plate (37.5 °C) at 40 × magnification. Percentages of morphologically normal and abnormal spermatozoa, as well as the viability, were evaluated on a phase-contrast microscope (100 × objective with emersion oil) after Eosin-Nigrosin staining. For both viability and morphology, 100 spermatozoa were evaluated.

Because of the coagulum bonobos can produce in the ejaculate, the ejaculate of the first bonobo (n1) was incubated for 60 min at body temperature before diluting in the two different mediums and gradually cooling to 4 °C. After this incubation period, a second evaluation of the ejaculate was performed, which revealed a significant quality loss. Therefore, we decided to remove this incubation step from our protocol and to dilute the ejaculate of the other bonobos (n2, n3, and n4) immediately after collection and the cooling process were initiated.

### Semen cryopreservation

For cryopreservation, we compared a one-step commercial Freezing Medium (IrvineScientific, Santa Ana, USA) (one-step medium) and a two-step in-house prepared Uppsala diluter (two-step medium) [33]. The one-step medium is successfully used for cryopreservation of human semen [44], whereas the two-step medium is widely used for cryopreservation in dogs [33]. When the volume allowed us, the semen sample was split into two equal volumes and randomly assigned to one of the cryopreservation methods. In one animal (n2) only the two-step medium was used because a critically low volume of semen was collected. The one-step medium was added in a ratio of 1:5. For the other half of the sample, the first step of the two-step medium was added in a ratio of 1:2.5. After that, both semen samples were gradually cooled to 4 °C in two hours using an ultra-dense styrofoam canine transport box with two cold packs (Minitube). Subsequently, for the samples cryopreserved in the two-step medium, the same volume of second medium was added, to have a final dilution of 1:5. Hereafter, the samples were manually divided in 0.25 ml straws before starting the cryopreservation procedure and kept in nitrogen vapors (at seven cm above the liquid nitrogen) for twenty minutes before they were plunged into the liquid nitrogen. The samples were cryopreserved based on volume, not based on concentration.

### Post-thaw evaluation

After 24 h in liquid nitrogen, two straws of each bonobo, one of each cryopreservation protocol, were thawed. These straws sat at room temperature for three seconds before being plunged into a warm water bath of 37 °C for 30 seconds. Following the thawing, the straws were dried and the semen was put in a pre-warmed (37 °C) 1.5 ml tube (Eppendorf). Motility was evaluated using an Integrated Semen Analysis System (ISAS®_v1_, Proiser R + D SL, Spain). Eosin-Nigrosin staining was performed to evaluate the semen viability and morphology.

## Data Availability

The datasets used and/or analyzed during the current study are available from the corresponding author on reasonable request.

## Abbreviations

AI: Artificial insemination
TYB: TEST-yolk buffer
IUCN Red List: International Union for Conservation of Nature’s Red List
EAZA: European Association of Zoos and Aquaria
EEP: Ex Situ Program
SSP: Species Survival Program
SIV: Simian immunodeficiency virus
SLTV: Simian T-cell lymphotropic virus
DNA: Deoxyribonucleic acid
V: Volt
ISAS: Integrated Semen Analysis System
TES: N-tris(hydroxymethyl)-2-aminoethane sulfonic acid
Tris: Tris(hydroxymethyl)-aminomethane
KMDA: Koninlijke Maatschappij voor dierkunde van Antwerpen
